# Effects of _L_-Cystine and _L_-Theanine Supplementation on the Common Cold: A Randomized, Double-Blind, and Placebo-Controlled Trial

**DOI:** 10.4061/2010/307475

**Published:** 2010-05-12

**Authors:** Shigekazu Kurihara, Takenori Hiraoka, Masahisa Akutsu, Eiji Sukegawa, Makoto Bannai, Susumu Shibahara

**Affiliations:** ^1^Research Institute for Health Fundamentals, Ajinomoto Co., Inc., Kanagawa 210-8681, Japan; ^2^Wellness Promotion Center, Ajinomoto Co., Inc., Kanagawa 210-8681, Japan

## Abstract

The common cold is one of the most frequent illnesses caused by viral infection. Recently, we have reported that oral administration of cystine and theanine (CT) to mice enhanced the humoral immune response associated with antibody production. Based on this mouse study, we investigated the effects of CT supplementation on the common cold in humans as a pilot study. A total of 176 healthy male volunteers were randomized to receive either placebo or CT (490 mg) tablets twice daily for 35 days. The incidence outcome was assessed using the definition in our laboratory based on questionnaires regarding cold symptoms. The incidence of subjects with colds during the trial was significantly lower in the CT group than in the placebo group, although the duration of the colds was not significantly different between the groups. These results suggest that CT supplementation may be useful for the prevention of the common cold.

## 1. Introduction

The common cold, an acute infection properly known as “cold syndrome,” is the most common human illness. The majority of cases of cold syndrome are acute infections of the upper respiratory tract, and its major cause is viral infection. From 30 to 50% of cases of cold syndrome are caused by rhinoviruses, and 10 to 15% are caused by coronaviruses [1]. Conventional methods of treatment use medications, such as analgesic agents and antihistamines, but these are only effective for the alleviation of symptoms, such as sneezing and runny nose [2]. Furthermore, antiviral agents, such as neuraminidase inhibitors, are believed to be effective; however, their application is currently limited to the influenza virus [1, 3–6]. Recently, Chinese medicine and dietary supplements have attracted attention as effective new methods for the treatment and prevention of cold syndrome [7]; for example, vitamin C; allysine, which is found in garlic; and the extract of the natural herb *Echinacea *[8–10]. However, there have also been reports indicating that the incidence of cold syndrome is unaffected by the above treatments [11, 12], and thus their effectiveness has yet to be clearly demonstrated.

In a previous experiment in mice, we revealed that the oral administration of _L_-cystine and _L_-theanine (CT) reinforced antigen-specific antibody production after antigen stimulation; these effects were possibly caused by the reinforcement of glutathione (GSH) synthesis and the humoral immune response [13]. Based on previous reports, the objective of this pilot study was to evaluate the effects of CT against cold syndrome in humans, using a double-blind comparative study. 

## 2. Methods

### 2.1. Procedure

This randomized, double-blind, and parallel-group comparative study with a placebo group as a reference was performed as a pilot study in accordance with the Declaration of Helsinki and after obtaining approval from all members of the Ajinomoto Co., Inc., Institutional Review Board (IRB), as organized by the executive director, the director of the quality assurance department, the general managers of research and development, and the divisional director of food business on December 17, 2001.

### 2.2. Subjects

The subjects were healthy male volunteers (for safety reasons and to avoid individual differences influenced by the estrous cycle) who are employees of Ajinomoto Co., Inc., excluding those with a history of or a current liver or kidney disease as well as those with critical illness who were not able to be diagnosed by a clinician. The subjects were randomly allocated into one of two groups using an allocation table: a group receiving placebo (P) and another group receiving cystine/theanine (CT). Both groups were studied at the same time. Before the test, we determined the number of subjects with allergy symptoms, the number of smokers, the number of subjects who caught a cold twice or more per year, and the number of subjects living with school-aged children, which are the family members most likely to catch a cold. When the subjects registered for the study, the investigator and the physician in charge provided the informed consent form, which was discussed and approved by the IRB. The content of the study was explained in detail, and the subjects provided their written voluntary consent.

### 2.3. Applied Dose

According to the third National Health and Nutrition Examination Survey that took place in the USA from 1988 to 1994, the average intake of _L_-cystine or _L_-cysteine from meals and supplements is 1000 mg/day. In males in the 51 to 70-year-old age group, the average intake among the subjects in the highest 1% of the group was 2200 mg/day [14]. Considering the safety data described above, the applied dose in the present study was set at 700 mg/day for _L_-cystine to remain within the range of the average daily intake of _L_-cystine or _L_-cysteine. The applied dose of _L_-theanine was calculated as 280 mg/day based on analyses in mice, which suggested that the most effective weight ratio of cystine  :  theanine = 5  :  2 [13]. Glycine was used as a placebo so that the subjects received the same source of nitrogen, and the applied dose was set at 980 mg/day.

### 2.4. Study Procedure

During the 5-week period from January 18, 2002 to February 21, 2002, the subjects were asked to take 2 tablets orally after breakfast and 2 tablets orally after dinner, with each tablet containing 175 mg of _L_-cystine plus 70 mg of _L_-theanine or 245 mg of glycine as a placebo (*ϕ*11 mm, Table 1) (a total of 4 tablets daily; total daily consumption of cystine, theanine, and glycine = 700 mg, 280 mg, and 980 mg, resp.). Each tablet was manufactured by Aliment Industry Co., LTD. (Yamanashi, Japan).

The onset of common cold symptoms as well as data regarding intake of the test meals during this 5-week period were recorded by the subjects on a daily basis as described below. 

The subjects performed a self-evaluation of 19 symptom items (“symptoms of the common cold”: runny nose, stuffy nose, sneezing, sore throat, cough, sputum, chills, fever, headache, joint pain, pain in the skin, and general malaise; “adverse events accompanying test meal ingestion”: nausea, vomiting, abdominal pain, diarrhea, loss of appetite, chest pain, and skin rash) which were graded according to 3 levels (+: presence of symptoms, ±: slight symptoms, and −: absence of symptoms). Data were recorded, and the subjects' temperatures were taken under the arm. The subjects were not restricted with regard to medical treatment during this period, such as taking medication for the common cold, but were asked to record any treatment received.

### 2.5. Data Processing and Statistical Analyses

In the analysis, the criteria used in the clinical test of anti-influenza virus agents were used as a reference [5, 6] because the period of the trial (January to February) corresponds to the season in which influenza virus is most prevalent in Japan. The symptoms recorded on the data sheets were converted to scores as follows: presence of symptoms (+) = 2, slight symptoms (±) = 1, and absence of symptoms (−) = 0. The items were classified into four predominant symptoms: (1) stuffy nose/runny nose/sneezing as nasal symptoms, (2) cough/sputum/sore throat as throat symptoms, (3) chills and fever as febrile symptoms, and (4) headache/joint pain/pain in the skin as pain symptoms. If the score for the predominant symptom was more than 3 of 6 for nasal, throat, or pain symptoms, the predominant symptom was considered to be present. We considered febrile symptoms to be present if the score was more than 2 of 4. If 3 of 4 predominant symptoms were observed on the same day or if the body temperature was above 37.0°C and two or more common cold symptoms (stuffy nose/runny nose/sneezing, cough/sputum/sore throat/chills/headache/joint pain/pain in the skin/general malaise) displayed a score of 2, the subject was considered to have the common cold (Table 2). Based on this definition, the number of subjects with incidences of the common cold during the 5-week period of the study was counted, and the two groups were compared using Fisher's exact test. When there was another incidence of the common cold, according to the above definition, after 3 or more days of recovery from an earlier cold, this was counted as a separate incident. The number of affected days was also counted and was compared between the two groups using Poisson regression analysis. In addition, the average duration of the colds and the number of subjects that experienced a body temperature above 37.0°C were also determined and were compared between the two groups using the *t*-test and Fisher's exact test, respectively. The incidences of individual symptoms of the cold and adverse events in the evaluation of safety were counted if the score had reached 2 at least once during the 35-day period of the trial, and the number was compared between the two groups using Fisher's exact test. All statistical analyses were performed using the Statistical Analysis System (SAS) version 8.2 (SAS Institute, Inc., Cary, NC, USA), and the results were considered as significant at *P* < .05.

## 3. Results

### 3.1. Background of the Subjects

The total number of subjects registered in this study was 176, and, as a result of the randomized allocation, 89 subjects were allocated into the CT group and 87 subjects were assigned to the P group. One subject in the CT group and one subject in the P group lost their data recording sheets, and one subject in the P group withdrew the informed consent. Therefore, the final analysis was performed using data from a total of 173 subjects, consisting of 88 subjects in the CT group and 85 subjects in the P group.  Table 3 shows the results of the background factor questionnaire, which was completed by the subjects prior to the study; there were no significant differences in background factors between the two groups. The tablet ingestion rate during the trial period indicates an approximately 90% ingestion rate in both groups, where ingestion of 70 tablets during the 5-week trial period was defined as 100% (Table 4). We counted the number of subjects in each group according to the ingestion rate, and there was no significant difference between the two groups (*P* = .357).

### 3.2. Effectiveness

**Figure 1:**
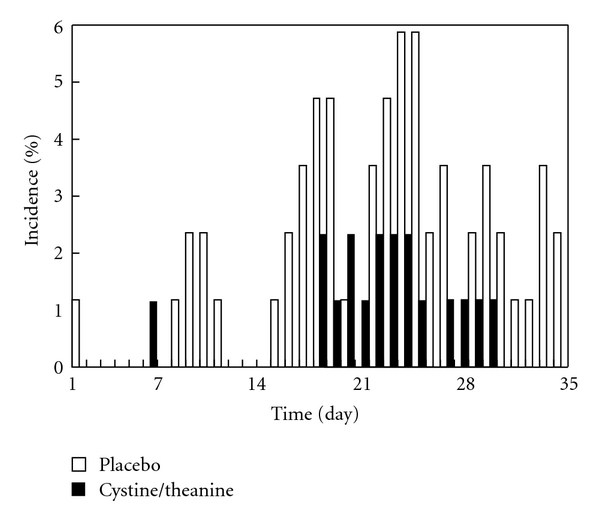
Incidence of laboratory-defined colds on each day during the 5-week trial period.

As shown in Figure 1, the incidence of the common cold in the CT group was lower than in the P group throughout the 5-week period of the trial. As shown in Table 5, the incidences of the common cold according to the definition set by our laboratory were 11.4% in the CT group and 27.1% in the P group, with that in the CT group being significantly lower than that in the P group (*P* = .011). The cumulative incidences during the trial period were 10 in the CT group and 29 in the P group (in the placebo group, four subjects had two and one subject had three separate instances of colds during the trial period); the CT group demonstrated a significantly reduced number of colds than the P group (*P* = .002). In addition, the cumulative numbers of days affected by the common cold were 18 days in the CT group and 59 days in the P group; the CT group demonstrated significantly fewer days affected by the cold than the P group (*P* = .002). The average duration of the colds was approximately 10% less in the CT group than that in the P group, although this difference was not significant. The proportion of subjects with body temperatures above 37°C was 18.2% in the CT group and 32.9% in the P group, with that in the CT group being significantly lower than that in the P group (*P* = .036). The average duration of a body temperature above 37°C was approximately 15% shorter in the CT group than in the P group, but the difference was not significant. In addition, a Poisson regression analysis was performed to look for correlations between the incidence of colds and the backgrounds of the subjects as well as to investigate any interactions between CT ingestion and the backgrounds of the subjects on the incidence of colds. However, no significant correlations or interactions were observed between the CT ingestion and the backgrounds of the subjects or the incidence of colds in the present trial (data not shown).

The results of the analyses regarding the incidences of each symptom in the trial period are shown in Table 6. For all items except pain in the skin, the incidences of symptoms were lower in the CT group than in the P group. Chills and fever in particular, included in the febrile symptoms, displayed significant decreases in incidence in the CT group (*P* = .029 and *P* = .003, resp.). The incidences of predominant symptoms other than febrile symptoms, such as runny nose (*P* = .057), sore throat (*P* = .089), and joint pain (*P* = .069), tended to be lower in the CT group than in the P group, although the differences were not statistically significant. The number of times that drugs to treat the common cold were taken during the trial period was 11 times in the CT group and 20 times in the P group (data not shown).

### 3.3. Adverse Events

The adverse events accompanying test meal ingestion were also examined. The incidences of diarrhea in the CT and P groups were 14.8% and 15.3%, respectively, which were not significantly different (Table 7). No other adverse events related to CT ingestion were observed.

## 4. Discussion

The present exploratory trial showed that continuous ingestion of CT significantly suppressed the incidence of the common cold compared to a placebo control group and also significantly reduced the incidence of chills and fever among individual symptoms. 

Ikematsu et al. investigated the safety of continuous ingestion of CoQ10 and asked subjects to self-evaluate the severity of common cold symptoms on a 4-level scale [15]. The results indicated that 24 of 85 subjects (28.2%) experienced incidences of common cold symptoms. By contrast, in a report on the prevention of upper respiratory tract infection using extract of North American ginseng containing poly-furanosyl-pyranosyl-saccharides by Predy et al., 10 types of common cold symptoms were scored on a 4-level scale, and the sum of the scores on 2 consecutive days over 14 was considered as an incidence of the common cold. In the placebo group, the incidence of colds was 22.8% during the 4-month trial period [16]. Considering the above findings as well as the differences in weather conditions and evaluation methods, the 27.1% incidence of colds (P group) in our study indicated that the definition of the common cold and the evaluation method used in this study were valid. 

Many studies have investigated the effectiveness of vitamin C in common cold suppression. Limited to those under physically stressed conditions, a meta-analysis of the effects of vitamin C indicated 50% suppression of the incidence of the common cold [17]. In addition, in the above-mentioned study by Predy et al., ingestion of the ginseng extract was shown to reduce the incidence of the common cold by approximately 56% [16]. Moreover, the extract of the natural herb *Echinacea* was reported to have a prophylactic efficacy of 55% or 58% on the incidence of common cold by two meta-analyses [18, 19]. By contrast, a study that employed continuous administration of *N*-acetylcysteine (NAC) indicated a 43% reduction in the incidence of influenza-like symptoms [20]. In the present study, the incidence of the common cold according to our definition was decreased by approximately 58% by CT ingestion. This result indicated that CT has a cold prevention effect with an efficacy similar to that of other nutrients that are considered to possess this effect. As previously mentioned, the majority of common colds are caused by viral infection, with rhinoviruses being the most common infectious agent [1]. These viruses are known to infect cells via intercellular adhesion molecule-1 (ICAM-1), which is a receptor on epithelial cells of the respiratory tract, and reduced GSH is known to inhibit the increased expression of ICAM-1 [21]. In our previous study in mice, we showed that the administration of CT significantly increases the amount of reduced GSH [13]. The above findings suggest that the increase in reduced GSH due to the ingestion of CT, which suppressed the expression of the virus receptor, led to prevention of the common cold and reduced the severity of cold symptoms.

The average duration of the colds was reduced by approximately 10%, although this reduction was not statistically significant. Thirty clinical studies were compared in a meta-analysis that was employed for the vitamin C study, and it was found that the duration of the colds was reduced by 8% in adults and 13.6% in children due to the ingestion of vitamin C [22]. Taking these results into consideration, we feel that another study of about the same scale or larger than this study will reveal a clear effect of CT on the consecutive number of days that individuals are affected by the common cold.

For individual symptoms of the common cold, significant reductions in febrile symptoms (chills and fever) were observed due to CT ingestion as well as a decreasing trend in the incidence of other predominant symptoms, for example, runny nose, sore throat, and joint pain. Riedel and Maulik reported that the increase in body temperature after lipopolysaccharide stimulation can be suppressed by administering a reducing agent, and they suggested the possibility that the increase in reduced GSH in response to the reducing agent suppressed fever [23]. NAC is known to show anti-inflammatory effects via GSH [24]. Reduced GSH is known to suppress the activation of NF-*κ*B, which is an inflammatory mediator at the time of rhinoviral infection [21]. These findings suggest that the suppression of individual symptoms of the common cold in the present study may have been due to the anti-inflammatory effect of increased GSH caused by CT ingestion. In this trial, there were some cases of observed symptoms but a lack of a cold during the trial. Consequently, we performed a Pearson correlation coefficient analysis between the incidence of colds during the trial and each symptom. The symptoms of chill (*r* = 0.694), fever (*r* = 0.663), and arthralgia (*r* = 0.610) showed strong correlations with the incidence of colds. In addition, the symptoms of cough (*r* = 0.467), runny nose (*r* = 0.458), and headache (*r* = 0.451) showed moderate correlations with the incidence of colds. These findings suggest that the definition of colds used in the present trial has a strong correlation with relatively severe symptoms.

In evaluating the safety of this study, we observed a relatively high incidence of diarrhea in the CT group as an adverse event. However, approximately the same incidence of diarrhea was observed in the P group, and therefore this effect is unlikely to be an adverse reaction specific to CT ingestion. In the present study, tablets were used in the form of a test meal. These tablets contained nondigestible substances, such as crystalline cellulose and dextrin, to maintain their shape, and these substances may have affected the digestive system. 

CT, which has been shown to reinforce GSH synthesis and antibody production in mice following antigen stimulation, was used in humans, and a significant inhibitory effect on the common cold has been observed. In a clinical study in humans, Miyagawa et al. reported that oral administration of CT improves antibody production in the elderly, with decreased immune function at the time of flu vaccination [25]. Moreover, Murakami et al. reported that CT ingestion by long-distance runners before a training camp suppressed the increase in blood neutrophil counts and the decrease in lymphocyte counts observed in control subjects after training [26]. Those results suggest that CT ingestion may improve the immunosuppression induced by aging in the elderly and by intense exercise in athletes. To determine whether this common cold inhibitory effect is due to the reinforcement of GSH synthesis and antibody production by CT ingestion, more detailed analyses are required using both animal and human subjects. The results of the present exploratory trial showed that CT is an effective and safe drug as a food ingredient to suppress the common cold, and we anticipate its use as a drug or food ingredient for cold prevention and symptom relief in the future.

## 5. Conclusion

A randomized, placebo-controlled 5-week trial indicated that CT supplementation significantly reduced the incidences of colds and of fever symptoms, but had no apparent effect on the duration of colds. These results suggest that CT supplementation may be useful for the prevention of the common cold.

## Figures and Tables

**Table 1:** Components of each tablet.

	Cystine/Theanine	Placebo
_L_-Cystine	175	—
_L_-Theanine	70	—
Glycine	—	245
Microcrystalline cellulose	100	100
Dextrin	114	115
Micronized silicon dioxide	2.5	10
Glycerine fatty acid ester	32.5	25
Aspartame	1	—
Vanilla flavor	5	5

Total	500	500

**Table 2:** Criteria of laboratory-defined colds.

Incidence of each main symptom	
Nasal	Total score/day ≧ 3/6
Throat	
Pain	
Febrile	Total score/day ≧ 2/4

Incidence of colds	

Number of the positive main symptom ≧ 3/day	
or	
Body temperature ≧37°C and number of the cold symptom score	
of 2 ≧ 2/day	

**Table 3:** Characteristics of the volunteers.

Characteristic	Cystine/Theanine group (*n* = 88)	Placebo group (*n* = 85)	*P*-value
Age (years; mean ± SE)	39.8 ± 1.2	39.6 ± 1.2	.906^a^
Number of the volunteers			
– Allergic predisposition (%)	32 (36.4)	36 (42.4)	.440^b^
– Smokers (%)	22 (25.0)	24 (28.2)	.731^b^
– Colds more than twice per year (%)	57 (64.8)	56 (65.9)	1.000^b^
– Living with school-age children (%)	30 (34.1)	24 (28.2)	.418^b^

^a^The *t*-test was used for comparisons between groups.

^b^Fisher's exact test was used for comparisons between groups.

**Table 4:** Compliance rate of each tablet during the 5-week trial period.

	Cystine/Theanine group (*n* = 88)	Placebo group (*n* = 85)	*P*-value^a^
Level of compliance (%; mean ± SE)	88.5 ± 1.7	91.4 ± 1.1	—
– Number of volunteers (%)			
90~100%	56 (63.6)	58 (68.2)	
70~90%	23 (26.1)	22 (25.9)	.357
<70%	9 (10.2)	5 (5.9)	

^a^Cochran-Mantel-Haenszel ANOVA test was used for comparisons between groups.

**Table 5:** Incidence and duration of laboratory-defined colds during the 5-week trial period.

	Cystine/Theanine group (*n* = 88)	Placebo group (*n* = 85)	*P*-value
Laboratory-defined colds			
– Number of volunteers (%)	10 (11.4)	23 (27.1)	.011^a^
– Number of incidence	10	29	.002^b^
– Cumulative days of incidence	18	59	.002^b^
– Average duration (mean ± SE)	1.8 ± 0.8	2.0 ± 1.1	.883^c^
Temperature, ≧37.0°C			
– Number of volunteers (%)	16 (18.2)	28 (32.9)	.036^a^
– Average duration (mean ± SE)	1.7 ± 0.2	2.0 ± 0.2	.182^c^

^a^Fisher's exact test was used for comparisons between groups.

^b^Poisson regression analysis was used for comparisons between groups.

^c^The *t*-test was used for comparisons between groups.

**Table 6:** Cold symptoms during the 5-week trial period.

Symptom		Cystine/Theanine group (*n* = 88)	Placebo group (*n* = 85)	*P*-value^a^
Nasal	Runny nose	24 (27.3)	35 (41.2)	.057
	Nasal congestion	19 (21.6)	22 (25.9)	.593
	Sneeze	16 (18.2)	23 (27.1)	.203

Throat	Sore throat	19 (21.6)	29 (34.1)	.089
	Cough	17 (19.3)	23 (27.1)	.280
	Sputum	16 (18.2)	19 (22.4)	.572

Fever	Chill	10 (11.4)	21 (24.7)	.029
	Fever	9 (10.2)	24 (28.2)	.003

Pain	Headache	15 (17.0)	20 (23.5)	.345
	Arthralgia	7 (8.0)	15 (17.6)	.069
	Dermatalgia	2 (2.3)	1 (1.2)	1.000

General malaise		13 (14.8)	21 (24.7)	.126

^a^Fisher's exact test was used for comparisons between groups.

**Table 7:** Adverse events during the 5-week trial period.

Adverse events (%)	Cystine/Theanine group (*n* = 88)	Placebo group (*n* = 85)	*P*-value^a^
Nausea	5 (5.7)	6 (7.1)	.764
Vomiting	1 (1.1)	4 (4.7)	.205
Abdominal pain	8 (9.1)	9 (10.6)	.802
Diarrhoea	13 (14.8)	13 (15.3)	1.000
Anorexia	4 (4.5)	8 (9.4)	.243
Chest pain	2 (2.3)	0 (0.0)	.497
Skin rash	0 (0.0)	0 (0.0)	—

^a^Fisher's exact test was used for comparisons between groups.
